# Author Correction: Control of electronic topology in a strongly correlated electron system

**DOI:** 10.1038/s41467-022-34314-5

**Published:** 2022-10-31

**Authors:** Sami Dzsaber, Diego A. Zocco, Alix McCollam, Franziska Weickert, Ross McDonald, Mathieu Taupin, Gaku Eguchi, Xinlin Yan, Andrey Prokofiev, Lucas M. K. Tang, Bryan Vlaar, Laurel E. Winter, Marcelo Jaime, Qimiao Si, Silke Paschen

**Affiliations:** 1 Institute of Solid State Physics, Vienna University of Technology, 1040 Vienna, Austria; 2 High Field Magnet Laboratory (HFML-EMFL), Radboud University, 6525 ED Nijmegen, The Netherlands; 3 Los Alamos National Laboratory, Los Alamos, NM 87545 USA; 4 Department of Physics and Astronomy, Rice Center for Quantum Materials, Rice University, Houston, TX 77005 USA

**Keywords:** Topological matter, Electronic properties and materials, Phase transitions and critical phenomena

**Correction to**: *Nature Communications* 10.1038/s41467-022-33369-8, published online 29 September 2022

The original version of this Article contained an error in the last paragraph of the Introduction, which incorrectly read ‘Upon tuning the Weyl-Kondo semimetal [12] Ce_3_Bi_4_Pd_3_ [11,13] by magnetic field we observe a continuous suppression of the giant topological response associated with the material’s Kondo-driven Weyl nodes, and the annihilation of the nodes.’ The correct version replaces this sentence with ‘Upon tuning Ce_3_Bi_4_Pd_3_ [12], a Weyl-Kondo semimetal [11–13], by magnetic field we observe a continuous suppression of the giant topological response associated with the material’s Kondo-driven Weyl nodes, and the annihilation of the nodes.’

This has been corrected in the PDF version of the Article.

