# Supplementary material for: Control of electronic topology in a strongly correlated electron system
Source: Nat Commun. 2022 Sep 29;13:5729. doi: 10.1038/s41467-022-33369-8 (PMC9523050; doi:10.1038/s41467-022-33369-8)
Supplement: Supplementary file 1 — Supplementary Information [file 41467_2022_33369_MOESM1_ESM.pdf]

Supplementary Information

Control of electronic topology in a strongly correlated electron system

Sami Dzsaber[1], Diego A. Zocco[1], Alix McCollam[2], Franziska Weickert[3], Ross McDonald[3], Mathieu Taupin[1], Gaku Eguchi[1], Xinlin Yan[1], Andrey Prokofiev[1], Lucas M. K. Tang[2], Bryan Vlaar[2], Laurel E. Winter[3], Marcelo Jaime[3], Qimiao Si[4], and Silke Paschen[1,*]

[1]*Institute of Solid State Physics, Vienna University of Technology, 1040 Vienna, Austria*

[2]*High Field Magnet Laboratory (HFML-EMFL), Radboud University, 6525 ED Nijmegen, The Netherlands*

[3]*Los Alamos National Laboratory, Los Alamos, NM 87545, USA*

[4]*Department of Physics and Astronomy, Rice Center for Quantum Materials, Rice University, Houston, TX 77005, USA*

Supplementary Note 1: Summary of Weyl-Kondo characteristics of $\mathbf{Ce_3Bi_4Pd_3}$

$Ce_3Bi_4Pd_3$ crystallizes in the cubic $Y_3Sb_4Au_3$ phase of space group $I\bar{4}3d$, which is noncentrosymmetric and nonsymmorphic[1], and preserves time reversal symmetry as evidenced by muon spin rotation[2]. It thus fulfills the necessary conditions to be a (nonmagnetic) Weyl semimetal. Indeed, density functional theory (DFT) identified a number of Weyl nodes in its electronic structure, albeit more than 100 meV away from the Fermi energy[2]. This is consistent with the expectation that even though DFT cannot capture the correlation physics, it does reflect the symmetry constraints.

Furthermore, $Ce_3Bi_4Pd_3$ is a heavy fermion compound with a single ion Kondo temperature of 13 K as estimated from specific heat[1]. It becomes fully Kondo coherent only below about 3 K as shown by a magnetoresistance analysis[1]. As the temperature is lowered into the Kondo coherent regime, the material—still governed by the same space-group constraint—hosts Kondo-generated heavy Weyl quasiparticles, with Weyl nodes near the Fermi energy.

There are two key signatures of Weyl-Kondo nodes in the Kondo coherent state: (i) a giant spontaneous (zero field) Hall effect and an associated (equally giant) even-in-field continuation of it in finite fields (Fig. 1e), and (ii) an electronic specific heat coefficient $\Delta C/T$ that is linear in T^2, i.e. $\Delta C/T = \Gamma T^2$, and is associated with the linear dispersion of the Weyl nodes (Fig. 1f).

Both emerge only in the Kondo coherent state[1,2] and are thus clearly Kondo driven. The giant magnitude of the spontaneous Hall effect was attributed to Weyl nodes—where the Berry curvature diverges—being essentially pinned to the Fermi energy[2]. The giant value of the slope Γ reveals that the Weyl quasiparticles have velocities v^* that are renormalized by three orders of magnitude with respect to values typical for noninteracting Weyl or Dirac semimetals (as shown in[1,3], $\Gamma \propto (1/v^*)^3$). Thus, the Kondo interaction appears to create—from the Weyl nodes present in the noninteracting bandstructure—extremely flat Weyl-Kondo cones in the immediate vicinity to the Fermi level, which is commensurate with theoretical expectations for a pertinent model[3].

The spontaneous Hall effect was also shown to have nonlinear current-voltage characteristics and to display a second harmonic response under AC current drive[2]. Other contributions such as contact misalignment[2], skew scattering (Supplementary Note 8), or multiband effects (Supplementary Note 9) were ruled out to play any significant role (see also Ref. [2]). Also spurious extrinsic effects can be discarded because (i) the spontaneous Hall effect is reproducible from sample to sample (Fig. S8 of Ref. [2]), (ii) it exhibits a systematic Hall angle dependence (Fig. S9 of Ref. [2]), (iii) nonlinear-in-current DC, as well as 0ω and 2ω AC contributions occur as expected and set in at the same temperature (Figs. 2D-F, S6, S7 and Table S1 of Ref. [2]), and (iv) the spontaneous Hall signal emerges as the material becomes Kondo coherent (Fig. 1D of Ref. [2]).

Two comments are due: Firstly, because the Weyl bands of $Ce_3Bi_4Pd_3$ are flat bands (renormalized by three orders of magnitude compared to the noninteracting case), ARPES lacks the resolution needed to resolve them. The linear dispersion is instead detected by $\Delta C/T \propto T^2$ (see above). Secondly, magnetotransport signatures of the chiral anomaly are expected to be suppressed by the strongly reduced quasiparticle velocities (equally by three orders of magnitude compared to the noninteracting case). The spontaneous Hall effect plays the analogous role in that it probes the Berry curvature distribution.

Both above-mentioned key signatures (i and ii), as well as an associated third one, an odd-in-field anomalous Hall effect (Fig. 1d and Supplementary Fig. 6 top row), are successively suppressed with increasing magnetic field (Fig. 1d-f) and vanish, in the $T = 0$ limit, at a critical field B_{c1} (Fig. 4a, red symbols).

Supplementary Note 2: Semimetallic ground state of $Ce_3Bi_4Pd_3$ in zero magnetic field

In zero magnetic field, $Ce_3Bi_4Pd_3$ is a Weyl-Kondo semimetal[2]. The semimetallic nature of the ground state is also evidenced by the temperature dependence of the normal Hall coefficient R_H (the slope of the linear-in-field contribution to the antisymmetrized Hall resistivity, see Supplementary Note 7), which saturates at low temperatures to a constant value (see Fig. 1b and $\tilde{R}_0$ in Fig. 2d). In a simple one-band model, R_H is inversely proportional to the charge carrier concentration n. A saturating R_H thus corresponds to a saturating n. In view of the small value of n (of the order of $10^{26}\,\mathrm{m}^{-3}$), this is a characteristic of a semimetal. Note that this behavior is also seen in the $Ce_3Bi_4Pd_3$ sample studied in[4] (Supplementary Fig. 1), even though an insulating ground state is claimed in that work.

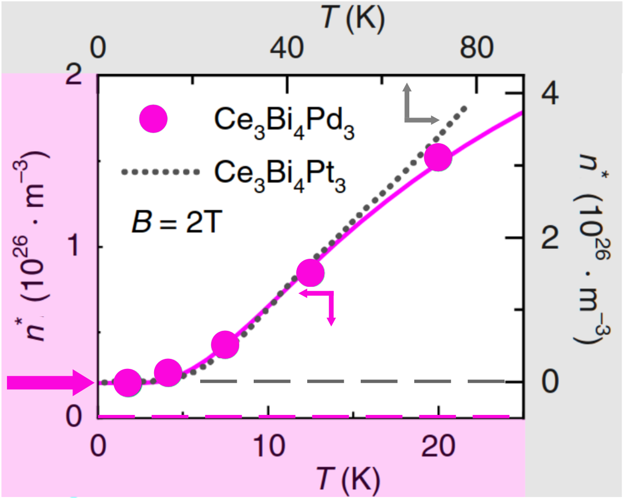

Supplementary Fig. 1: Temperature-dependent charge carrier concentrations of $Ce_3Bi_4Pd_3$ and $Ce_3Bi_4Pt_3$. Charge carrier concentration n^* of $Ce_3Bi_4Pd_3$ (pink, bottom and left axes) and of $Ce_3Bi_4Pt_3$ (grey, top and right axes), as extracted from the (antisymmetrized) Hall resistivity at 2 T in a one-band model. For $Ce_3Bi_4Pd_3$, n^* saturates to a finite value in the low-temperature limit (pink arrow), consistent with a semimetallic ground state. By extension, the Hall coefficient is not thermally activated in the relevant low-temperature regime. For $Ce_3Bi_4Pt_3$, by contrast, n^* saturates to zero (on this linear scale), consistent with the Kondo insulating ground state of this compound. Figure adapted from[4].

Supplementary Note 3: Cartoon of the correlated bandstructure of $Ce_3Bi_4Pd_3$ under magnetic field tuning

Taking all results of the present work together allows us to draw a cartoon of how the correlated electronic bandstructure of $Ce_3Bi_4Pd_3$ changes under the action of the magnetic field (Supplementary Fig. 2). Note that this is a strongly simplified sketch, which ignores realistic dispersions, the multiplicity of Weyl nodes etc. Ab initio-based many-body calculations will be needed to refine this picture.

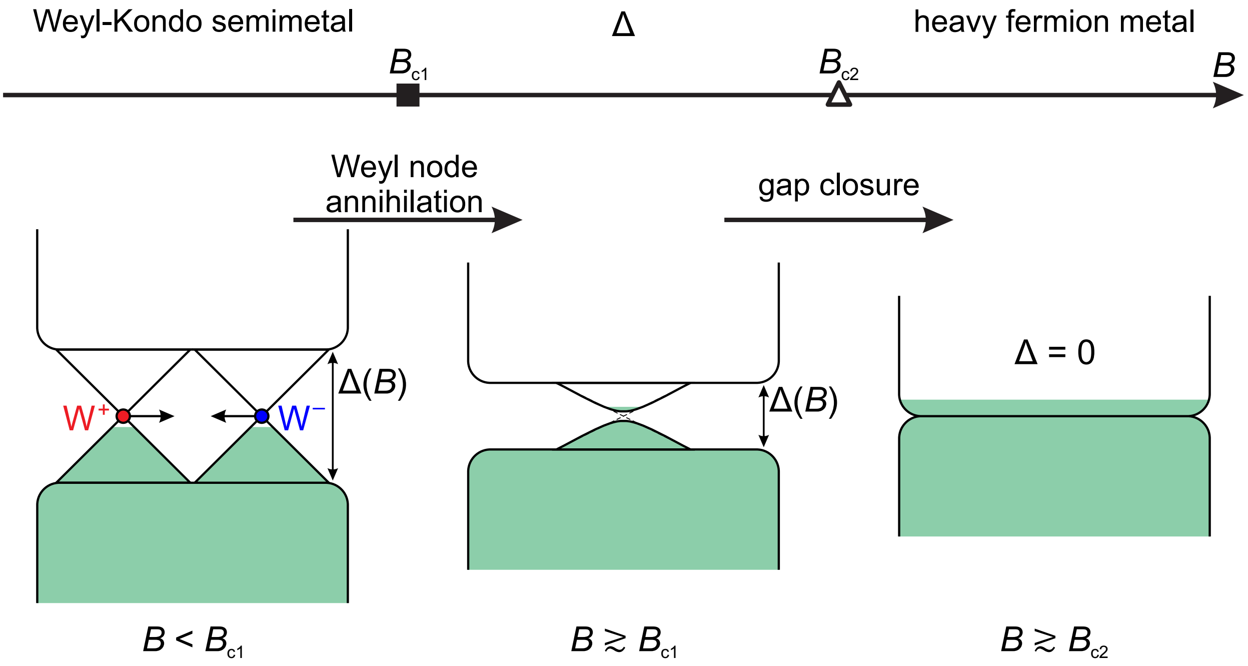

Supplementary Fig. 2: Cartoon of the correlated electronic bandstructure of $Ce_3Bi_4Pd_3$ under magnetic field tuning. At low fields, a Weyl (W^+) and an anti-Weyl node (W^-) are situated within a Kondo insulator gap [$\Delta(B)$], slightly above the Fermi energy (the green shading represents the electron filling). The momentum-space separation between the nodes decreases with increasing magnetic field (arrows), but their energy is unchanged. At a first critical magnetic field (B_{c1}), the Weyl nodes meet in momentum space and annihilate, which gaps out the Weyl dispersion. At a second critical magnetic field (B_{c2}), the Kondo insulator gap is closed and the system becomes a heavy fermion metal.

Supplementary Note 4: Evidence for a two-stage transition from electrical resistivity

As pointed out in the main text, the rather drastic features seen at B_{c1} and B_{c2} in the field-dependent Hall resistivity isotherms (Fig. 1c) leave clear signatures also in the temperature- and field-dependent electrical resistivity (Fig. 1a,c). In Supplementary Fig. 3a we show the relevant field range of $\rho_{xx}(B)$ data at 120 mK. A rather pronounced change of the magnetoresistance character is observed at B_{c1}. The magnetoresistance of Kondo systems is typically negative and sizeable, in agreement with the broadened step-like resistance decrease obtained by Schlottmann from a Bethe-Ansatz solution of the Coqblin-Schrieffer model ("Schlottmann scaling")[5]. Such behavior is seen both in the low-field range below B_{c1} and in the intermediate-field range between B_{c1} and B_{c2} (see shaded orange and green curves in Supplementary Fig. 3a). The "double decay" shape separated by B_{c1} naturally suggests that the magnetoresistance of $Ce_3Bi_4Pd_3$ is governed by two different scales, one of the Weyl-Kondo semimetal below B_{c1} and one of the intermediate phase

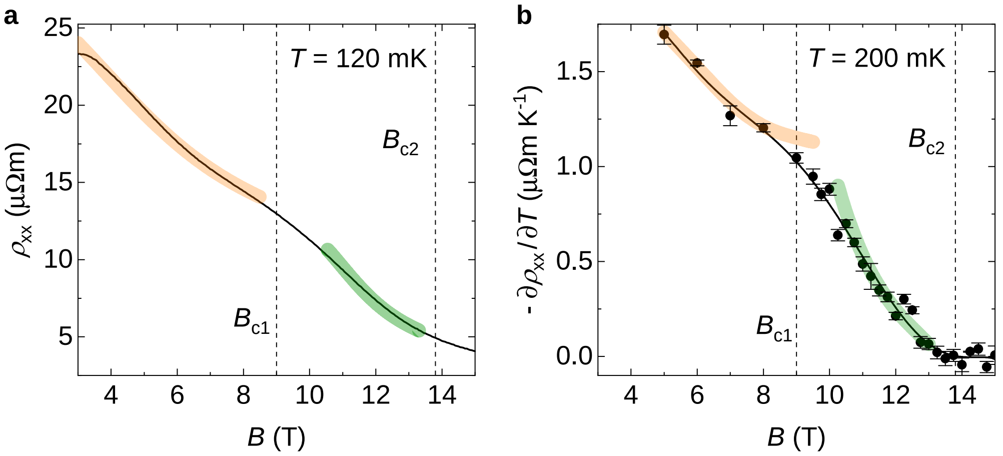

Supplementary Fig. 3: Field-dependent electrical resistivity. (**a**) Electrical resistivity vs magnetic field at 120 mK, together with Schlottmann fits[5] (see text). Their purpose is solely to indicate what type of magnetoresistance might be expected in a Kondo system. It is clear that the entire curve cannot be attributed to a Kondo system with a single energy scale. (**b**) Temperature derivatives (full symbols) of iso-field $\rho_{xx}(T)$ curves, taken at 200 mK, defined as the slopes of the linear fits in Supplementary Fig. 4. The error bars represent the standard errors of the slopes of these fits. The lines are guides to the eyes (they have also Schlottmann shape, see shaded lines). Signatures at the critical fields B_{c1} and B_{c2} are clearly revealed.

between B_{c1} and B_{c2}. The signature at B_{c2} is more pronounced in iso-field $\rho_{xx}(T)$ curves, as shown next. In Supplementary Fig. 3b we plot the temperature derivative of these curves, taken at 200 mK, vs magnetic field (see Supplementary Fig. 4 for how the derivatives were obtained). Here, not only the transition at B_{c1} is clearly resolved, but also that at B_{c2}. To conclude, the field- and temperature-dependent electrical resistivity, with clear signatures at B_{c1} and B_{c2}, underpins the presence of the three phases discussed in the main text.

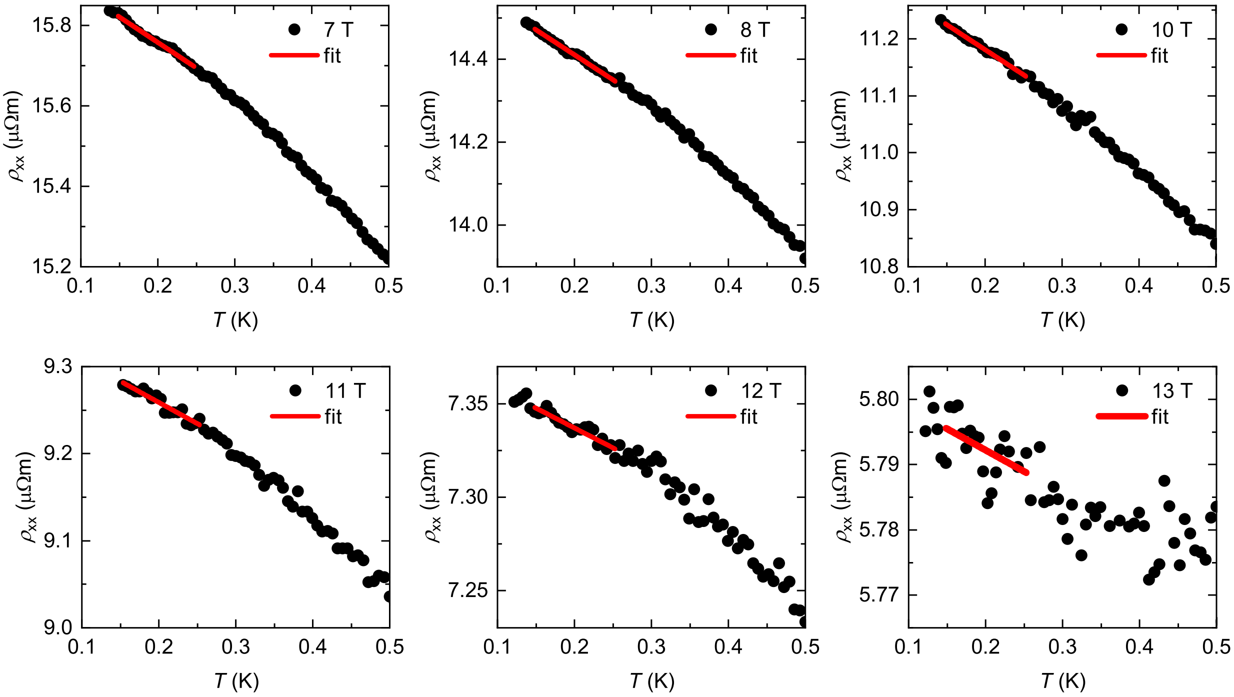

Supplementary Fig. 4: Temperature dependent iso-field electrical resistivity curves. The red lines are linear fits to the data (full symbols) in the range (200 ± 50) mK. Their slopes correspond to the local derivative of $\rho(T)$ at 200 mK and are plotted vs field in Supplementary Fig. 3.

Supplementary Note 5: Specific heat

In a Weyl-Kondo semimetal, the electronic contribution to the temperature-dependent specific heat varies as $C = \Gamma T^3$, with $\Gamma = 7\pi^2 k_B^2/(\hbar v^\star)^3$ (Ref. [3]), as experimentally observed in $Ce_3Bi_4Pd_3$ (Ref. [1]). The strongly renormalized quasiparticle velocity $v^\star$ boosts the electronic ΓT^3 term to the point that it even overshoots the Debye βT^3 term of the lattice[1].

To track the evolution of the Weyl-Kondo semimetal phase in $Ce_3Bi_4Pd_3$ with magnetic field,

we measured the temperature dependent specific heat $C_{\mathrm{Ce}}(T)$ of the compound at several fixed magnetic fields. In a first step, we determine the electronic contribution to the measured data by subtracting the specific heat $C_{\mathrm{La}}(T)$ of the nonmagnetic reference compound $La_3Bi_4Pd_3$. In a second step, to isolate the temperature-dependent part, we subtract a trivial Sommerfeld offset γ in the $(C_{\mathrm{Ce}} - C_{\mathrm{La}})/T$ data of $\gamma = 209$ mJ/(mol-Ce K^2), which is in good agreement with $\gamma = 200$ mJ/(mol-Ce K^2) measured elsewhere[1]. We observe that the $\Delta C/T = \Gamma T^2$ behavior seen at $B = 0$ is successively suppressed with increasing magnetic field, until it is no longer discernible for $B = 9$ T (Fig. 1f).

Supplementary Note 6: Even-in-field Hall resistivity

As discussed in Ref.[2] and summarized in Supplementary Note 1, the spontaneous Hall effect is the key signature of a Weyl-Kondo semimetal in zero magnetic field. In finite magnetic field, it finds continuation in an even-in-field contribution $\rho_{xy}^{\mathrm{even}}(B)$ (Supplementary Fig. 5), that can be obtained by symmetrizing the Hall resistivity (after eliminating contact misalignment contributions, see Supplementary Information Sect. I of Ref.[2]). $\rho_{xy}^{\mathrm{even}}(B)$ cannot be induced by the magnetic field. Instead, it is—just as the spontaneous Hall effect—due to the odd-in-momentum Berry curvature divergences at the Weyl nodes of a TRS preserving (nonmagnetic) Weyl semimetal. As this contribution cannot be confused with any other Hall contribution (such as multiband effects, magnetization-related anomalous Hall effect, etc., see Supplementary Information Sect. I of Ref.[2]), it is the key signature of the Weyl-Kondo semimetal in finite magnetic field. The role of the magnetic field is to tune the Weyl-Kondo semimetal phase and ultimately suppress it, thereby consecutively weakening $\rho_{xy}^{\mathrm{even}}(B)$ and finally suppressing it as the Weyl nodes annihilate, as shown in Fig. 1e and discussed in the main text.

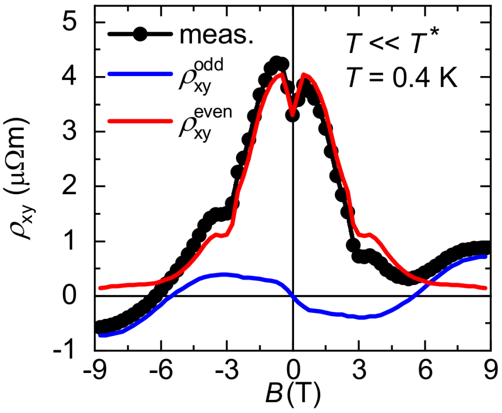

Supplementary Fig. 5: Hall resistivity of $Ce_3Bi_4Pd_3$ at 0.4 K. The magnetic field-dependent DC Hall resistivity $\rho_{xy}(B)$ consists of an odd-in-B ($\rho_{xy}^{\mathrm{odd}}(B)$, blue) and an even-in-$B$ ($\rho_{xy}^{\mathrm{even}}(B)$, red) component. The former is "normal" in the sense that it is induced by the magnetic field. It can be further decomposed into a linear-in-field normal Hall effect, and a nonlinear anomalous Hall effect induced by the time reversal symmetry (TRS)-breaking applied magnetic field. The even-in-B component, which cannot be induced by the magnetic field, is the finite-field continuation of the spontaneous (zero field) Hall effect. It is due to the odd-in-momentum Berry curvature divergences at the Weyl nodes of a TRS preserving (nonmagnetic) Weyl semimetal. As this contribution cannot be confused with any other Hall contribution (such as multiband effects, magnetization-related anomalous Hall effect, etc.), it is the key signature of the Weyl-Kondo semimetal. From Ref.[2].

Supplementary Note 7: Analysis of the normal Hall resistivity data

In the linear response sense, the Hall coefficient R_{H} is defined as the initial slope of the Hall resistivity $\rho_{xy}(B)$, i.e., $R_{\mathrm{H}} = \lim_{B\to 0} \partial\rho_{xy}(B)/\partial B$. In the case of a driving magnetic field B_0, it may still be defined as $R_{\mathrm{H}}(B_0) = \partial\rho_{xy}(B)/\partial B$ at $B = B_0$. In heavy fermion systems that undergo a magnetic field-tuned quantum phase transition, it has been experimentally demonstrated by cross-field experiments that it is the local derivative $\partial\rho_{xy}(B)/\partial B$ that corresponds to the Hall coefficient[6,7].

In experiments where a magnetic field simultaneously drives the phase transition and is used to measure the Hall resistivity, $R_{\mathrm{H}}(B)$ follows a step-like function that sharpens as the temperature

is lowered, and can be fitted with a function

$$R_{\mathrm{H}}(B) = R_{\infty} - (R_{\infty} - R_0)\left[1 + \left(\frac{B}{B^*}\right)^{p}\right]^{-1} \tag{1}$$

where R_0 and R_∞ are the Hall coefficients of the initial ($B \to 0$) and final phases ($B \to \infty$), respectively, B^* is the magnetic field at the transition, and p is associated with the width of the transition. The total Hall resistivity is then given by

$$\rho_{xy} = \int R_{\mathrm{H}}(B)dB \ . \tag{2}$$

The Hall resistivity of $Ce_3Bi_4Pd_3$ (Fig. 1c) has two-step nature; therefore three phases must be considered (low field: 0; intermediate field: 1; high field: 2), each with a constant Hall coefficient (R_0, R_1, and R_2) defined as the field derivative of the Hall resistivity $\rho_{xy}(B)$. The total fitting function concatenates the information from the slopes of $\rho_{xy}(B)$ from each phase, resulting in the fitting function

$$R_{\mathrm{H}}(B) = R_2 - (R_2 - R_{\mathrm{H},1})\left[1 + \left(\frac{B}{B_2^*}\right)^{p_2}\right]^{-1} \tag{3}$$

where

$$R_{\mathrm{H},1}(B) = R_1 - (R_1 - R_0)\left[1 + \left(\frac{B}{B_1^*}\right)^{p_1}\right]^{-1} \ . \tag{4}$$

The characteristic widths of the transitions between consecutive phases (ΔB_1 and ΔB_2) correspond to the full width at half maximum (FWHM) of the second derivatives of the calculated $\rho_{xy}(B)$ curves.

Fits at two selected temperatures are shown in the main part (Fig. 2a,b). Further fits are shown in Supplementary Fig. 6. All parameters obtained from these fits are displayed in Fig. 2c-f.

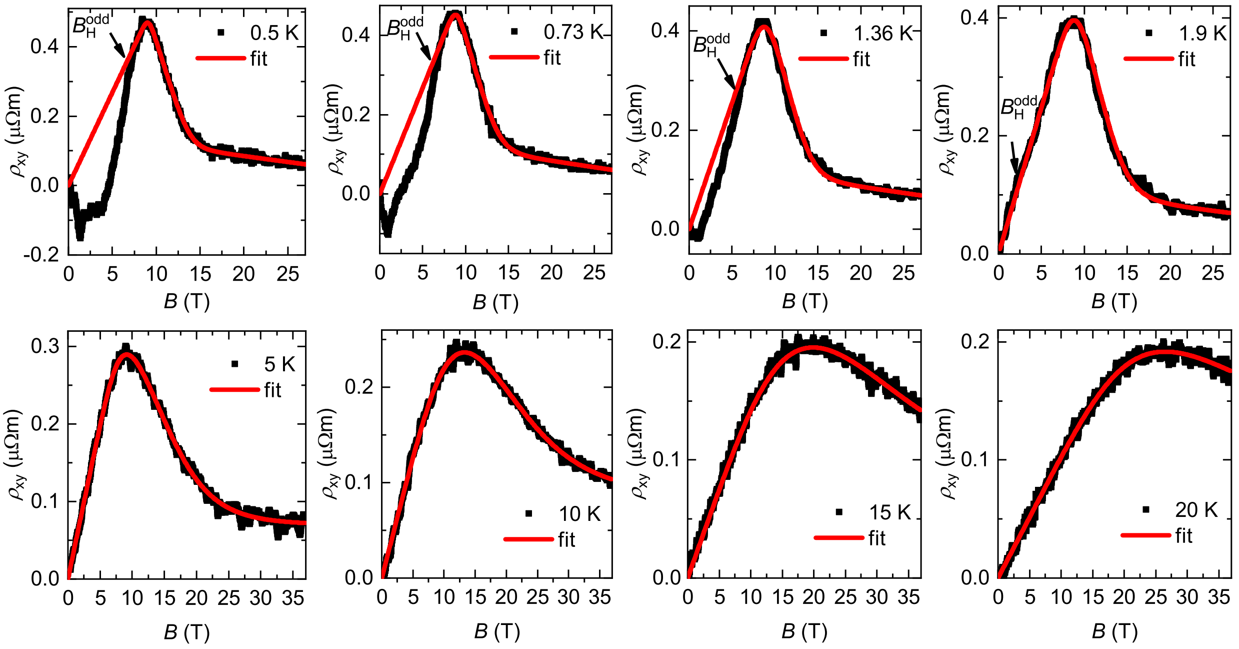

Supplementary Fig. 6: Two-stage crossover fits to the Hall resistivity. Hall resistivity data for various temperatures between 0.5 and 20 K (black symbols), and the corresponding two-stage transition fits (red lines) using Supplementary Eqs. (1-4). Below 1.36 K, the fit range is between 8.4 and 27 T, to avoid influence of the Berry curvature-related (odd-in-B) anomalous Hall effect (negative contribution superimposed to the linear-in-B normal Hall effect discussed also in Ref. [2]). Arrows indicate the field $B_{\mathrm{H}}^{\mathrm{odd}}$ above which the deviation from the initial linear behavior (red line) drops below 5 %. These fields are plotted as temperature scales $T_{\mathrm{H}}^{\mathrm{odd}}(B)$ in Fig. 4a. With increasing temperature, the crossovers broaden and move to higher fields. Thus, at 5 K and above, fits were done up to 37 T to capture as much of the crossover behavior as possible. At 15 K and above, the second transition has moved out of the measured magnetic field range, and thus a two-stage crossover fit loses meaning. All fitted parameters are displayed in Fig. 2c-f.

Supplementary Note 8: Anomalous Hall effect

Heavy fermion compounds in their Kondo incoherent regime at elevated temperatures frequently exhibit a significant anomalous Hall signal due to skew scattering[8]. In the Kondo coherent regime at low temperatures, by contrast, this contribution is known to become very small[6,8,9]. The skew scattering-driven anomalous Hall effect is frequently estimated by $\rho_A(B) \sim \rho(B)M(B)$ (Ref. [8]). This is, however, only valid if the field dependence of ρ is dominated by that of the scattering time[10]. If the Fermi surface undergoes an abrupt change, as is the case in the Kondo destruction scenario of heavy fermion metals[6] and as we observe here for $Ce_3Bi_4Pd_3$ at B_{c1} and B_{c2}, the field dependence of ρ may be dominated by that of the charge carrier concentration. In this situation, the expression $\rho_A(B) \sim M(B)$ (Refs.[11,12]) is more appropriate. Thus, in Supplementary Fig. 7, we plot $\rho_{xy}(B)$ together with the magnetization $M(B)$ (Ref. [4]), scaled by γ_2 to fit $\rho_{xy}(B)$ at low ($B < B_{c1}$), intermediate ($B_{c1} < B < B_{c2}$), and high ($B > B_{c2}$) fields. It is clear that none of the characteristic features of $\rho_{xy}(B)$ is seen in $M(B)$. Thus, skew scattering is not the cause of the observed magnetic field dependence of ρ_{xy} in $Ce_3Bi_4Pd_3$.

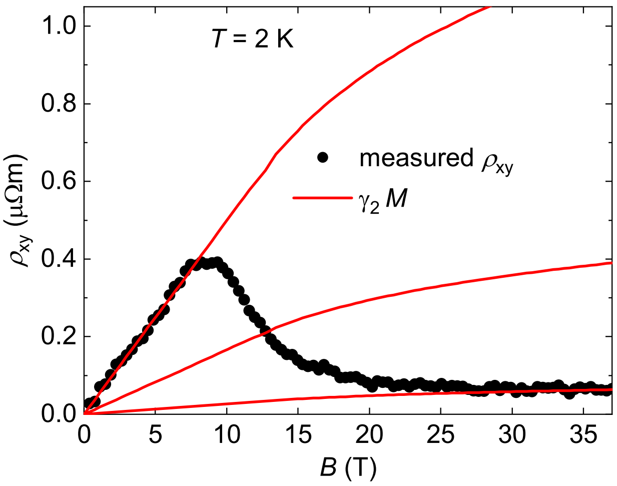

Supplementary Fig. 7: Estimation of skew scattering contribution to Hall effect from magnetization. Field-dependent Hall resistivity at 2 K, together with magnetization data at 1.5 K taken from Ref. [4] and scaled by γ_2 to match ρ_{xy} at low, intermediate, and high fields (see text). The fact that $\gamma_2 M(B)$ does not reproduce the shape of $\rho_{xy}(B)$ reveals that the anomalous Hall effect due to skew scattering is unimportant in $Ce_3Bi_4Pd_3$.

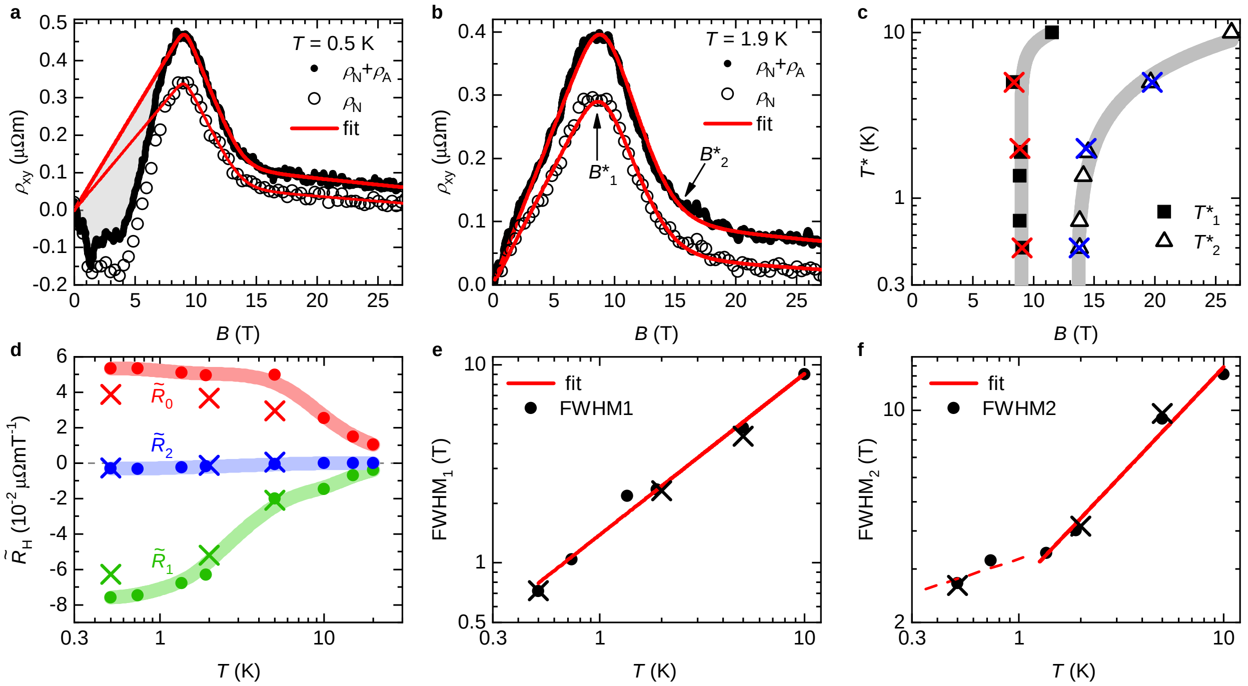

Supplementary Fig. 8: Estimation of skew scattering contribution to Hall effect from magnetization and electrical resistivity. Analysis of the two-crossover behavior as presented in Fig. 2, redone under the (unrealistic, see text) assumptions that, firstly, the field dependence of the electrical resistivity is due to skew scattering only and that thus the anomalous Hall effect is given by $\rho_A(B) = \gamma_2 \rho(B) M(B)$ and that, secondly, the prefactor γ_2 assumes the maximum possible value. (**a,b**) As-measured Hall resistivity isotherms (full black points) and data with the (hypothetical) skew scattering contribution ρ_A subtracted (open symbols), along with the respective two-transition fits. (**c-f**) Fit parameters obtained for the as-measured (full circles) and (hypothetical) skew scattering corrected data (crosses). The fact that the key fit parameters quantifying the crossovers (T^* and FWHM) are essentially unchanged shows that even the maximum estimated anomalous Hall contribution does not have any appreciable effect on the Hall crossovers.

In the next step we ignore the above conclusion and nevertheless use $\rho_A(B) = \gamma\, \rho(B)\, M(B)$ (Ref. [8]) as an estimate of the absolute maximum skew scattering contribution. We furthermore use $\gamma = 0.08$ K/T ($\gamma = 0.05$ Ce/μ_B when M is in units of μ_B/Ce), which is the upper limit for Ce-based heavy fermion materials[8]. We then subtract $\rho_A(B)$ from our data and redo the two-crossover fit at several representative temperatures (Supplementary Fig. 8). The results show that

the skew scattering contribution to the initial Hall coefficient is at most $\sim 20\%$ (Supplementary Fig. 8d). Importantly, neither the crossover positions nor their widths change considerably (Supplementary Fig. 8c,e-f). We conclude that even in the unlikely event that skew scattering is present in the $\rho_{xy}(B)$ data this would not contribute appreciably to the crossover behavior observed in $Ce_3Bi_4Pd_3$.

Supplementary Note 9: Hall effect from multiple bands

To investigate whether the pronounced nonlinearity of the Hall resistivity $\rho_{xy}(B)$ might, alternatively, be a multiple band effect, we have performed an effective two-band analysis using a recently established robust scheme[13]. We perform the analysis on a representative $\rho_{xy}(B)$ curve at $T = 1.9$ K, in three different fit ranges (see Supplementary Fig. 9), to test up to which field a two-band model can capture the observed behavior. All fit parameters are given in Supplementary Table 1.

We see that even the fit up to 11 T has very poor quality, showing that a two-band model cannot reproduce the sharp bending at B_{c1}. Also the parameters obtained for this fit (one electron and

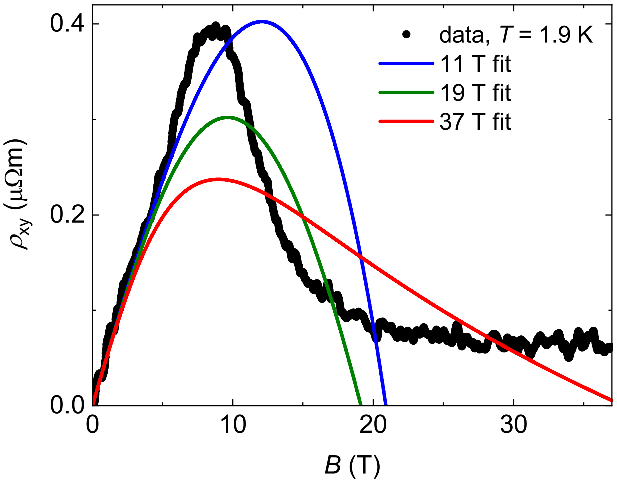

Supplementary Fig. 9: Estimation of contribution to Hall effect from multiband effects. Two-band analysis of the Hall resistivity vs field isotherm of $Ce_3Bi_4Pd_3$ at 1.9 K, using the robust analysis scheme of Ref. [13]. The three curves represent fits from zero field up to 11, 19, and 37 T, respectively, to test whether in any of these field ranges the experimental $\rho_{xy}(B)$ curve can be reproduced. The failure shows that the pronounced nonlinearity of $\rho_{xy}(B)$ cannot be due to two-band (or multiband) effects.

Supplementary Table 1: Fit parameters for two-band analysis done in Supplementary Fig. 9. All values are rounded up to the first digit, and thus, must be regarded as approximate values. A positive (negative) sign of the mobility denotes a hole-like (electron-like) band.

parameters	11 T	19 T	37 T	unit
n_1	$9 \cdot 10^{23}$	$7 \cdot 10^{25}$	$1 \cdot 10^{27}$	$1/m^3$
n_2	$9 \cdot 10^{23}$	$1 \cdot 10^{24}$	$1 \cdot 10^{23}$	$1/m^3$
μ_1	-2000	-40	-3	cm^2/Vs
μ_2	2000	500	1000	cm^2/Vs

one hole band with $|n_1| \approx |n_2| \approx 1 \times 10^{18}\,\text{cm}^{-3}$, $|\mu_1| \approx |\mu_2| \approx 2000\,\text{cm}^2/\text{Vs}$) appear rather unrealistic. Extending the field range does not bring any improvement. Thus, we conclude that it is highly unlikely that the observed nonlinearity is due to two- or multiband effects (even in the generic multiband situation, frequently two bands are found to dominate). The fact that the feature in $\rho_{xy}(B)$ at B_{c1} coincides with the anomalies in $\rho_{xx}(B)$ and $\partial\rho_{xx}/\partial T(B)$ (Supplementary Fig. 3B) as well as with all other temperature scales reported in Fig. 4a supports, instead, that it is due to a crossover between phases with different Fermi volume.

Supplementary Note 10: Analysis of torque magnetometry data

Angular-dependent magnetic torque measurements were obtained for $Ce_3Bi_4Pd_3$ and $Ce_3Bi_4Pt_3$ single crystals in pulsed fields up to 65 T at the NHMFL-LANL facility at Los Alamos. Piezoresistive levers of dimensions $120\,\mu$m $\times$ $50\,\mu$m $\times$ $4\,\mu$m and unloaded resonant frequency of 250 to 300 kHz were used. In order to keep the response fast in the few ms of rising magnetic fields, small samples were cut from previously characterized single crystals, resulting in the loss of the crystallographic alignment.

For cubic, magnetically isotropic materials, a linear magnetization results in zero magnetic torque[14]. Indeed, a sizable torque signal develops in $Ce_3Bi_4Pd_3$ and $Ce_3Bi_4Pt_3$ only near the characteristic fields B_τ (Fig. 3c,d). The angular dependence of the amplitude of the torque signal obtained at 0.5 K is displayed in Supplementary Fig. 10a for $Ce_3Bi_4Pd_3$ and Supplementary Fig. 10b for $Ce_3Bi_4Pt_3$. For both compounds we found that the magnetic torque anisotropy is dominated by

a sin(4Θ+α) component, as expected for magnetically anisotropic systems with cubic symmetry[15]. For $Ce_3Bi_4Pd_3$, the lower characteristic magnetic energy scale allowed us to obtain a large set of data points in pulsed fields up to 15 T (black dots). Full 65 T shots were performed then for selected angles (white dots). For this case, the angular dependence can be described by a superposition of a 4Θ and a 2Θ components, the former twice as large than the latter (ratio 4Θ/2Θ $\sim$ 2). We attribute the smaller 2Θ component to shape anisotropy effects[15].

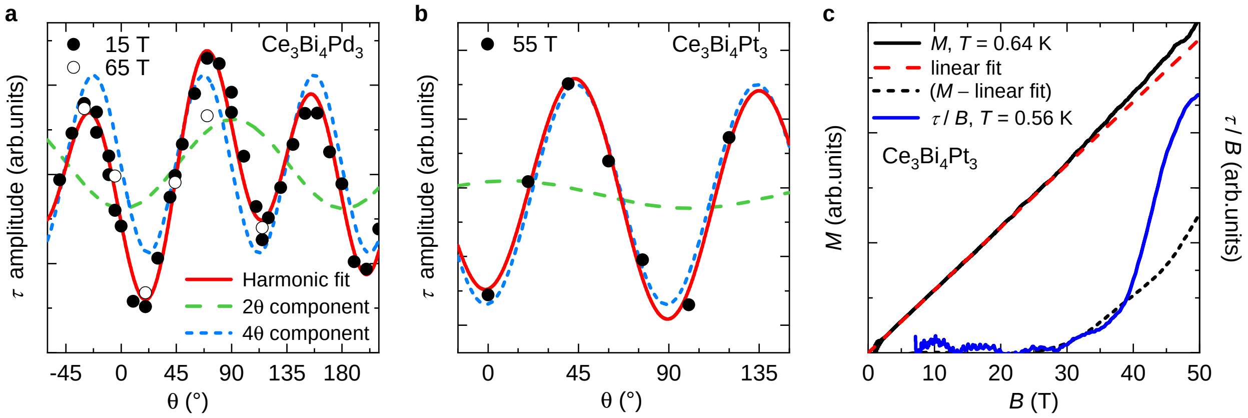

Supplementary Fig. 10: Torque magnetometry of $Ce_3Bi_4Pd_3$ and $Ce_3Bi_4Pt_3$. (**a**) Angular dependence of the torque signal of $Ce_3Bi_4Pd_3$ at 0.5 K, obtained for magnetic field sweeps up to 15 T (black dots) and 65 T (white dots). The red solid line is the best fit to the 15 T data, and it corresponds to the superposition of a dominant 4Θ component (blue dotted line) and a smaller 2Θ contribution (green dashed line). (**b**) Similar data for $Ce_3Bi_4Pt_3$, with the torque amplitudes obtained at 55 T and 0.5 K. (**c**) High-field magnetization (black curve, left axis) at 0.64 K and magnetic torque divided by applied magnetic field (blue curve, right axis) of $Ce_3Bi_4Pt_3$ at 0.56 K. Above 30 T, the magnetization departs from linear-in-B behavior (red dotted line, linear fit to the data below 30 T). The difference is plotted as black dotted curve on the right axis.

For $Ce_3Bi_4Pt_3$, the finite torque signal can only be accessed above 30 T. For this reason, fewer points of the angular dependence of the torque signal amplitude at 55 T are displayed in Supplementary Fig. 10b. Nevertheless, a good fit to the data can also be achieved with a superposition of 4Θ and 2Θ components (4Θ/2Θ $\sim$ 8). A full curve of magnetic torque divided by applied magnetic field is displayed in Supplementary Fig. 10c (blue solid line), alongside the measurement of magnetization obtained at a similar temperature (black solid line). Above 30 T, the magnetization

departs from linear-in-B behavior (red dotted line, linear fit to the data below 30 T). The difference is plotted as a black dotted curve on the right axis. The onset of nonlinearity in $M(B)$ thus coincides with the appearance of a sizeable torque signal.

Supplementary Note 11: Fermi and non-Fermi liquid behavior

At high fields (sufficiently far above B_{c2}) and low temperatures (below T_{FL}) the electrical resistivity of $Ce_3Bi_4Pd_3$ shows Fermi liquid behavior, $\rho = \rho_0 + AT^2$. This is evidenced by two sets of measurements: (i) iso-field temperature-dependent electrical resistivity measurements in a dilution refrigerator in fields up to $B = 15$ T (Supplementary Fig. 11a-d), and (ii) isothermal magnetic field sweeps (Fig. 1b) taken at a high-field laboratory (Methods) and converted into iso-field temperature dependences in fields up to 24 T (Supplementary Fig. 11e-g). The residual resistivity ρ_0 and the A coefficient extracted from fits to these data are shown in Supplementary Fig. 11h,i. Even at the largest field of 24 T, the A coefficient is still above 1 $\mu\Omega$cm/K^2. Using the Kadowaki–Woods ratio $A/\gamma^2 = 10^{-5}\mu\Omega\mathrm{cm}(\mathrm{mol\,K/mJ})^2$ this corresponds to a Sommerfeld coefficient of 316 mJ/(mol K^2), which corresponds to a mass renormalization of at least 2 orders of magnitude. Thus, the Kondo effect is still at play. The influence of the Kondo insulating phase in the vicinity of B_{c2} on $\rho(T)$ is discussed in the main text. It leads to a deviation from the divergence of the A coefficient indicated by the high-field data (Supplementary Fig. 11h, full line) and negative A values at $B < B_{c2}$. Also the continuous increase of the residual resistivity ρ_0 with decreasing field across B_{c2} (Supplementary Fig. 11i) reflects a transition into a Kondo insulating phase.

To investigate whether the enhancement of the A coefficient upon approaching B_{c2} from the high-field side is due to a quantum critical point at B_{c2}, we measured the electrical resistivity at 15 T in the temperature range between 0.1 and 6 K (Fig. 3e, top and right axes). The measurement was done on a sample from the same batch as the sample of Supplementary Fig. 11 (and Fig. 3e, bottom and left axes, with residual resistivity $\rho_0 \approx 4\,\mu\Omega$m). Indeed, above the $\rho_{xx} \sim T^2$ behavior at the lowest temperatures, we observe a non-Fermi liquid $\rho_{xx} \sim T$ behavior, that persists up to $T_{NFL} = 4.5$ K. This temperature is close to the Kondo coherence temperature (Ref. [2]). This is a sizeable range of non-Fermi liquid behavior which, as such, further supports the evidence for

quantum criticality.

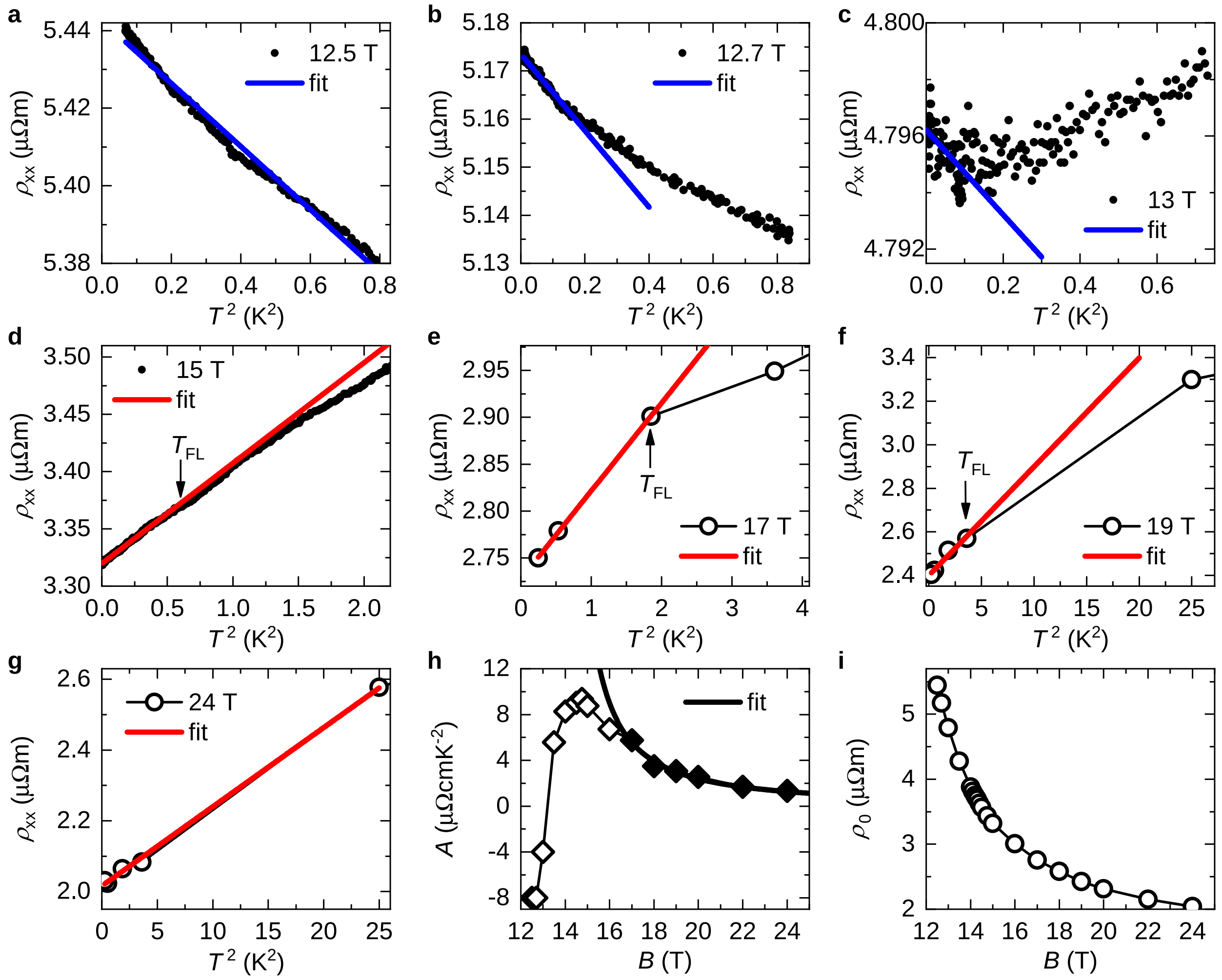

Supplementary Fig. 11: Analysis of Fermi liquid behavior in the electrical resistivity of $Ce_3Bi_4Pd_3$. (**a-g**) Electrical resistivity data plotted vs T^2 at various magnetic fields, with linear fits to determine the A coefficient, the residual resistivity ρ_0, and the onset temperature T_{FL}. (**h**) A coefficient vs applied magnetic field. The data above 16 T are well described by an $A \propto (B - B_{c2})^{-p}$ law with $B_{c2} = 13.8$ T and $p = 0.985$, suggesting that the effective mass diverges at a quantum critical point situated at B_{c2}. Below 16 T, the A coefficient deviates from this behavior to lower values, which indicates that contributions from the Kondo insulating phase start to mix in. (**i**) Residual resistivity ρ_0 vs applied magnetic field. The continued increase of ρ_0 with decreasing field even below B_{c2} is attributed to the Kondo insulating phase.

Supplementary Note 12: Sign change of Hall effect across Weyl node annihilation

Our experiments show that the (normal) differential Hall coefficient, which is the slope of the Hall resistivity isotherms (see Figs. 1d and 2a,b), changes sign at B_1^*. In the zero-temperature limit this change extrapolates to a discontinuous change (the FWHM vanishes in the $T = 0$ limit, see Fig. 2e) of the charge carrier concentration from a positive to a somewhat smaller negative value (Fig. 4b, assuming a single band model). At this transition the topological response vanishes (Fig. 4a, red symbols), which we attribute to a Weyl node annihilation (main text and Supplementary Note 1). A simple sketch of this process illustrates that a sign change may occur rather naturally (Supplementary Fig. 12).

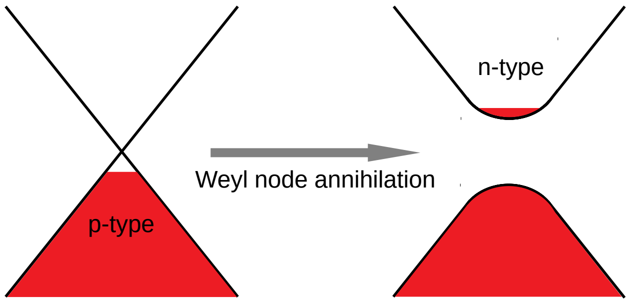

Supplementary Fig. 12: Sketch of Weyl node gapping out. When a Weyl and an anti-Weyl node meet in momentum space, they annihilate. This results in a gapping out of the nodes. The sketch shows only one Weyl node before (left) and after (right) the annihilation. In the Weyl-Kondo semimetal $Ce_3Bi_4Pd_3$ the Fermi pocket (before annihilation) is tiny due to the pinning of the Weyl nodes to the immediate vicinity of the Fermi energy by the Kondo effect. As a result, even a small gap will change the sign of the Hall effect. Our experiments show that the Weyl quasiparticles are p-type (holes in the lower Weyl cone). As the gap opens at B_{c1}, the lower band gets completely filled and left-over carriers populate the upper band, thus changing the system from being p-type to n-type.

Supplementary Note 13: Zeeman vs orbital effects of an applied magnetic field

The momentum space topology of electronic bands in crystalline solids depends on the material's symmetry, notably whether it preserves or breaks time reversal symmetry (TRS) and inversion symmetry (IS). The Weyl-Kondo semimetal $Ce_3Bi_4Pd_3$ studied here breaks IS (via its noncen-

trosymmetric crystal structure) but preserves TRS (see Supplementary Note 1 and Ref. [1]). In materials that preserve both symmetries, the application of a magnetic field may break TRS and thereby change the topological state of the material.

Generally, a magnetic field acts on magnetic moments via the Zeeman effect and on moving electrical charge via the orbital effect. As such it is intuitive that in materials where local magnetic moments play an important role the Zeeman effect will dominate whereas in materials with highly mobile charge carriers the orbital effect will be most prominent.

We are interested here in the influence of an applied magnetic field on Dirac and Weyl semimetals. Because the Zeeman effect can break TRS it is of particular interest.

Zeeman effect in noninteracting systems/systems without local moments. We first discuss the noninteracting case, taking the Dirac semimetal Cd_3As_2 as example. Using a combined DFT and effective low-energy model (based on $k \cdot p$ perturbation theory) it was shown[16] that the material's Dirac node splits into four Weyl nodes (two with chirality ± 1 as commonly assumed and two additional ones with chirality ± 2). The effect is subtle: Even a huge effective Zeeman field of 100 T creates only a tiny k-space splitting (about 0.5% of the Brillouin zone size). Furthermore, the created Weyl nodes are not degenerate in energy. Thus, though frequently evoked, the magnetic-field induced formation of a Weyl semimetal from a Dirac semimetal is not straightforward. To achieve a Zeeman field of 100 T in pure Cd_3As_2, a material strongly dominated by orbital effects (see below), seems highly unrealistic. In fact, alternative ways—magnetic doping or proximity to a ferromagnet—have been considered to boost the spin-exchange field[16,17].

Zeeman effect in heavy fermion compounds. In heavy fermion compounds, local magnetic moments are a natural ingredient of the system and thus a strong Zeeman effect does not need to be included artificially, as suggested in the Supporting Information of Ref. [3] and examined in Ref. [18]. In a Kondo lattice model for a simple nonsymmorphic and noncentrosymmetric crystal structure, the Zeeman effect was shown to move Weyl nodes—pinned to the Fermi energy—in momentum space until, at a continuous quantum phase transition, all Weyl nodes annihilate[18]. For the parameters selected in the model, this annihilation happens while the Kondo effect still operates.

Orbital effect in noninteracting systems. If Dirac points or Weyl nodes in noninteracting systems are situated near the Fermi energy (which may be achieved by doping), the low concentration of high-mobility carriers is expected to readily lead to quantum oscillations (just as in the case of clean topologically trivial semimetals). This has been seen in magnetotransport measurements of many noninteracting semimetals, both topological and topologically trivial. Some have been clearly evidenced to enter the quantum limit, where only the lowest Landau level is populated. The physics that may then happen is tunneling between left- and right-handed zeroth Landau levels, which is increasingly likely as the inverse magnetic length $1/l_{\mathrm{B}} = \sqrt{eB/\hbar}$, a measure of the width of the zeroth Landau level wavefunction in momentum space, becomes of the order of the Weyl node separation. The wavefunctions of the Weyl and anti-Weyl quasiparticles then mix and the topological response is lost[19]. This process happens at a finite distance of the Weyl nodes in momentum space. As such it does not correspond to a Weyl node annihilation, which happens only if a Weyl and an anti-Weyl node meet in momentum space. Furthermore, as the momentum space width of the zeroth Landau level wavefunction increases (linearly) with field, one expects a crossover and not a (quantum) phase transition, clearly distinct from the situation observed in the present work for $Ce_3Bi_4Pd_3$.

Another point, emphasized in Ref. [17], should also be noted. A large negative longitudinal magnetoresistance is a rather generic consequence of the quantum limit. It arises from the quenching of kinetic energy in the transverse directions and the associated suppression of backscattering in the effectively one-dimensional transport regime along the field direction. As concluded in Ref. [17], it should therefore not be taken as evidence for the chiral anomaly and thus for Weyl physics.

Orbital effect in heavy fermion compounds. In heavy fermion compounds, the heavy quasiparticles primarily originate from the f-electron local moments. As such, their orbital coupling to an external magnetic field is strongly suppressed (and the Zeeman coupling dominates). Moreover, the mass renormalization via the Kondo effect leads to a renormalization of the effective carrier mobility by the same amount. In $Ce_3Bi_4Pd_3$, the Kondo temperature is 13 K[1], about 1/1000th of a typical Fermi temperature in a noninteracting system. Thus, to reveal quantum oscillations in $Ce_3Bi_4Pd_3$ would require a 1000-fold higher magnetic field than what is needed in a corresponding

noninteracting system. Note that the low mobilities of heavy fermion systems are a direct consequence of the extreme correlation strength and do not evidence enhanced disorder. Consistent with this expectation is our result that, even at the highest field of 65 T (and the lowest temperature of 0.43 K) we do not see signs of quantum oscillations (Fig. 3a). Thus, contributions of orbital effects on the topological quantum phase transition we observe at 9 T can be safely discarded.

Supplementary Note 14: Comparison with weakly-interacting materials

Weakly interacting Dirac and Weyl semimetals have been explored quite extensively. The purpose of this section is to help appreciate that they behave qualitatively differently from the strongly correlated Weyl-Kondo semimetal $Ce_3Bi_4Pd_3$. We first summarize the effect of magnetic field on two such systems as examples.

$(Pb_{1-x}Sn_x)_{1-y}In_yTe$. In Ref. [20] evidence is reported for a topological metallic phase in $(Pb_{1-x}Sn_x)_{1-y}In_yTe$ that persists over a finite pressure range. $Pb_{0.75}Sn_{0.25}Te$ samples show an insulator–metal–insulator (I–M–I) transition as function of pressure (with the two critical pressures p_1 and p_2); in $(Pb_{0.5}Sn_{0.5})_{1-y}In_y$ samples only the first I–M transition (at p_1) could be experimentally accessed. In the metallic (high mobility, very low carrier concentration) regime, quantum oscillations are seen, consistent with tiny Fermi pockets. As discussed above (Supplementary Note 13) this evidences the importance of the orbital magnetic field effect. When pressure is set close to the I–M transition but in the I phase, magnetic field can be used to tune the system into the metallic phase. This gives rise to large negative (transverse and longitudinal) magnetoresistance which, in conjecture with density functional theory (DFT) calculations, was interpreted as evidence for Weyl nodes. Whether the ferroelectric distortion, which is assumed in the DFT calculations but not resolved in the actual materials, and/or the Zeeman effect play significant roles in the proposed creation of Weyl nodes from Dirac points across p_1 remains to be clarified. No data were yet provided for a Weyl node annihilation.

TaAs and TaP. In Ref. [19] high-field electrical resistivity measurements on TaAs are reported. At low fields (up to 7.5 T), quantum oscillations are visible. The approximate field independence of the resistivity ρ_{zz} (along the field direction, perpendicular to the Landau level quantization plane)

at intermediate fields is argued to evidence the chiral anomaly in a system with Weyl nodes and an inter-nodal scattering rate dominated by short-range impurity scattering. The increase of ρ_{zz} at even higher fields (around 50 T) is attributed to a gap formation due to the mixing of the left- and right-handed zeroth Landau level wavefunctions as described above (Supplementary Note 13). A simple tight-binding model for TaAs revealed[19] that the Zeeman effect is essentially negligible. Only for very large g factors (10), very large Weyl node separations ($0.15\pi/a$), and very large fields (65 T) an appreciable effect (10%) on the gap size (opened by the orbital effect) is seen. In Ref. [21], hysteretic behavior was observed at the quantum limit, but its origin remains to be clarified. In TaP the situation is very similar[22], the only difference being that one pair of Weyl nodes in TaP remains ungapped up to the highest measured fields.

Also other (candidate) Dirac and Weyl semimetals show evidence for the dominance of orbital effects: the above discussed Cd_3As_2[16,17] (Supplementary Note 13), as well as GdPtBi[23], $ZrTe_5$[24,25], ZrSiS[26], elemental Te[27–29], etc.

Similar contrast between strongly correlated and weakly correlated topology also applies for other control parameters. For instance, in sequences considered to be driven by increasing spin-orbit coupling strength, a Weyl-Kondo semimetal to Kondo insulator transition was observed in $Ce_3Bi_4(Pd_{1-x}Pt_x)_3$ with increasing x[1], whereas in the series of weakly interacting compounds NbP – TaP – TaAs, ARPES could only detect a relatively modest change in Weyl node separation (by 0.07 $Å^{-1}$ across the whole series)[30]. The ability to continuously vary the magnetic field and thus the Zeeman coupling, as opposed to discrete steps in such substitution studies, was crucial for our investigation and makes this technique particularly powerful.

Another topic investigated are transitions between phases of different broken symmetry that, by consequence, also feature different topological properties. Examples are CeSbTe[31] and $Co_3Sn_2S_2$[32,33]. In the latter ARPES data together with DFT calculations were taken as evidence for Weyl nodes being present in the ferromagnetic state below the Curie temperature $T_C = 176\,K$ but absent in the paramagnetic state above it[32], and/or for Dirac loops above T_C and ferromagnetic Weyl loops below T_C[33]. In these cases, it is not the topological state as such that is controlled, but the topologically trivial "background" that changes from being paramagnetic to ferromagnetic as

temperature is lowered. This is distinct from the effect we have discovered here: a genuine Weyl node annihilation in a qualitatively unchanged background.

In summary, we are not aware of any published evidence for a genuine (Zeeman tuned) annihilation of Weyl nodes in a topological quantum phase transition.

Supplementary References

1. Dzsaber, S., Prochaska, L., Sidorenko, A., Eguchi, G., Svagera, R., Waas, M., Prokofiev, A., Si, Q. & Paschen, S. Kondo insulator to semimetal transformation tuned by spin-orbit coupling. *Phys. Rev. Lett.* **118**, 246601 (2017).
2. Dzsaber, S., Yan, X., Eguchi, G., Prokofiev, A., Shiroka, T., Blaha, P., Rubel, O., Grefe, S. E., Lai, H.-H., Si, Q. & Paschen, S. Giant spontaneous Hall effect in a nonmagnetic Weyl-Kondo semimetal. *Proc. Natl. Acad. Sci. U.S.A.* **118**, e2013386118 (2021).
3. Lai, H.-H., Grefe, S. E., Paschen, S. & Si, Q. Weyl-Kondo semimetal in heavy-fermion systems. *Proc. Natl. Acad. Sci. U.S.A.* **115**, 93 (2018).
4. Kushwaha, S. K., Chan, M. K., Park, J., Thomas, S. M., Bauer, E. D., Thompson, J. D., Ronning, F., Rosa, P. F. S. & Harrison, N. Magnetic field-tuned Fermi liquid in a Kondo insulator. *Nat. Commun.* **10**, 5487 (2019).
5. Schlottmann, P. Bethe-Ansatz solution of the ground-state of the SU $(2j+1)$ Kondo (Coqblin-Schrieffer) model: Magnetization, magnetoresistance and universality. *Z. Phys. B* **51**, 223 (1983).
6. Paschen, S., Lühmann, T., Wirth, S., Gegenwart, P., Trovarelli, O., Geibel, C., Steglich, F., Coleman, P. & Si, Q. Hall-effect evolution across a heavy-fermion quantum critical point. *Nature* **432**, 881 (2004).
7. Friedemann, S., Oeschler, N., Wirth, S., Krellner, C., Geibel, C., Steglich, F., Paschen, S., Kirchner, S. & Si, Q. Fermi-surface collapse and dynamical scaling near a quantum-critical point. *Proc. Natl. Acad. Sci. U.S.A.* **107**, 14547 (2010).
8. Fert, A. & Levy, P. M. Theory of the Hall effect in heavy-fermion compounds. *Phys. Rev. B* **36**, 1907 (1987).
9. Custers, J., Lorenzer, K., Müller, M., Prokofiev, A., Sidorenko, A., Winkler, H., Strydom, A. M., Shimura, Y., Sakakibara, T., Yu, R., Si, Q. & Paschen, S. Destruction of the Kondo effect in the cubic heavy-fermion compound $Ce_3Pd_{20}Si_6$. *Nat. Mater.* **11**, 189 (2012).

10. Nagaosa, N., Sinova, J., Onoda, S., MacDonald, A. H. & Ong, N. P. Anomalous Hall effect. *Rev. Mod. Phys.* **82**, 1539 (2010).

11. Arushanov, E., Kloc, C., Hohl, H. & Bucher, E. The Hall effect in β-$FeSi_2$ single crystals. *J. Appl. Phys.* **75**, 5106 (1994).

12. O'Handley, R. C. *The Hall effect and its applications, Ed. C. L. Chien and C. R. Westgate* (Plenum Press, New York, 1980).

13. Eguchi, G. & Paschen, S. Robust scheme for magnetotransport analysis in topological insulators. *Phys. Rev. B* **99**, 165128 (2019).

14. Michelutti, B. & Morin, P. Paramagnetic torque in cubic rare-earth compounds. *Phys. Rev. B* **46**, 14213 (1992).

15. Cullity, B. D. & Graham, C. D. *Introduction to Magnetic Materials, 2nd Ed.* (Wiley-IEEE Press, Hoboken, New Jersey, USA; ISBN 978-0-471-47741-9, 2009).

16. Baidya, S. & Vanderbilt, D. First-principles theory of the Dirac semimetal Cd_3As_2 under Zeeman magnetic field. *Phys. Rev. B* **102**, 165115 (2020).

17. Burkov, A. A. & Balents, L. Weyl semimetal in a topological insulator multilayer. *Phys. Rev. Lett.* **107**, 127205 (2011).

18. Grefe, S. E., Lai, H.-H., Paschen, S. & Si, Q. Extreme response of Weyl-Kondo semimetal to Zeeman coupling. *arXiv:2012.15841* (2020).

19. Ramshaw, B. J., Modic, K. A., Shekhter, A., Zhang, Y., Kim, E.-A., Moll, P. J. W., Bachmann, M. D., Chan, M. K., Betts, J. B., Balakirev, F., Migliori, A., Ghimire, N. J., Bauer, E. D., Ronning, F. & McDonald, R. D. Quantum limit transport and destruction of the Weyl nodes in TaAs. *Nat. Commun.* **9**, 2217 (2018).

20. Liang, T., Kushwaha, S., Kim, J., Gibson, Q., Lin, J., Kioussis, N., Cava, R. J. & Ong, N. P. A pressure-induced topological phase with large Berry curvature in $Pb_{1-x}Sn_xTe$. *Sci. Adv.* **3** (2017).

21. Zhang, Q. R., Zeng, B., Chiu, Y. C., Schönemann, R., Memaran, S., Zheng, W., Rhodes, D., Chen, K.-W., Besara, T., Sankar, R., Chou, F., McCandless, G. T., Chan, J. Y., Alidoust, N., Xu, S.-Y., Belopolski, I., Hasan, M. Z., Balakirev, F. F. & Balicas, L. Possible manifestations of the chiral anomaly and evidence for a magnetic field induced topological phase transition in the type-I Weyl semimetal TaAs. *Phys. Rev. B* **100**, 115138 (2019).

22. Zhang, C.-L., Xu, S.-Y., Wang, C. M., Lin, Z., Du, Z. Z., Guo, C., Lee, C.-C., Lu, H., Feng, Y., Huang, S.-M., Chang, G., Hsu, C.-H., Liu, H., Lin, H., Li, L., Zhang, C., Zhang, J., Xie, X.-C., Neupert, T.,

Hasan, M. Z., Lu, H.-Z., Wang, J. & Jia, S. Magnetic-tunnelling-induced Weyl node annihilation in TaP. *Nat. Phys.* **13**, 979–986 (2017).

23. Hirschberger, M., Kushwaha, S., Wang, Z., Gibson, Q., Liang, S., Belvin, C. A., Bernevig, B. A., Cava, R. J. & Ong, N. P. The chiral anomaly and thermopower of Weyl fermions in the half-Heusler GdPtBi. *Nat. Mater.* **15**, 1161 (2016).

24. Chen, R. Y., Chen, Z. G., Song, X.-Y., Schneeloch, J. A., Gu, G. D., Wang, F. & Wang, N. L. Magnetoinfrared spectroscopy of Landau levels and Zeeman splitting of three-dimensional massless Dirac fermions in $ZrTe_5$. *Phys. Rev. Lett.* **115**, 176404 (2015).

25. Zheng, G., Zhu, X., Liu, Y., Lu, J., Ning, W., Zhang, H., Gao, W., Han, Y., Yang, J., Du, H., Yang, K., Zhang, Y. & Tian, M. Field-induced topological phase transition from a three-dimensional Weyl semimetal to a two-dimensional massive Dirac metal in $ZrTe_5$. *Phys. Rev. B* **96**, 121401 (2017).

26. VanGennep, D., Paul, T. A., Yerger, C. W., Weir, S. T., Vohra, Y. K. & Hamlin, J. J. Possible pressure-induced topological quantum phase transition in the nodal line semimetal ZrSiS. *Phys. Rev. B* **99**, 085204 (2019).

27. Hirayama, M., Okugawa, R., Ishibashi, S., Murakami, S. & Miyake, T. Weyl node and spin texture in trigonal tellurium and selenium. *Phys. Rev. Lett.* **114**, 206401 (2015).

28. Ideue, T., Hirayama, M., Taiko, H., Takahashi, T., Murase, M., Miyake, T., Murakami, S., Sasagawa, T. & Iwasa, Y. Pressure-induced topological phase transition in noncentrosymmetric elemental tellurium. *Proc. Natl. Acad. Sci. U.S.A.* **116**, 25530–25534 (2019).

29. Zhang, N., Zhao, G., Li, L., Wang, P., Xie, L., Cheng, B., Li, H., Lin, Z., Xi, C., Ke, J., Yang, M., He, J., Sun, Z., Wang, Z., Zhang, Z. & Zeng, C. Magnetotransport signatures of Weyl physics and discrete scale invariance in the elemental semiconductor tellurium. *Proc. Natl. Acad. Sci. U.S.A.* **117**, 11337–11343 (2020).

30. Liu, Z. K., Yang, L. X., Sun, Y., Zhang, T., Peng, H., Yang, H. F., Chen, C., Zhang, Y., Guo, Y., Prabhakaran, D., Schmidt, M., Hussain, Z., Mo, S.-K., Felser, C., Yan, B. & Chen, Y. L. Evolution of the Fermi surface of Weyl semimetals in the transition metal pnictide family. *Nat. Mater.* **15**, 27 (2016).

31. Schoop, L. M., Topp, A., Lippmann, J., Orlandi, F., Müchler, L., Vergniory, M. G., Sun, Y., Rost, A. W., Duppel, V., Krivenkov, M., Sheoran, S., Manuel, P., Varykhalov, A., Yan, B., Kremer, R. K., Ast, C. R. & Lotsch, B. V. Tunable Weyl and Dirac states in the nonsymmorphic compound CeSbTe. *Sci. Adv.* **4**, eaar2317 (2018).

32. Liu, D. F., Xu, Q. N., Liu, E. K., Shen, J. L., Le, C. C., Li, Y. W., Pei, D., Liang, A. J., Dudin, P., Kim,

T. K., Cacho, C., Xu, Y. F., Sun, Y., Yang, L. X., Liu, Z. K., Felser, C., Parkin, S. S. P. & Chen, Y. L. Topological phase transition in a magnetic Weyl semimetal. *Phys. Rev. B* **104**, 205140 (2021).

33. Belopolski, I., Cochran, T. A., Liu, X., Cheng, Z.-J., Yang, X. P., Guguchia, Z., Tsirkin, S. S., Yin, J.-X., Vir, P., Thakur, G. S., Zhang, S. S., Zhang, J., Kaznatcheev, K., Cheng, G., Chang, G., Multer, D., Shumiya, N., Litskevich, M., Vescovo, E., Kim, T. K., Cacho, C., Yao, N., Felser, C., Neupert, T. & Hasan, M. Z. Signatures of Weyl fermion annihilation in a correlated Kagome magnet. *Phys. Rev. Lett.* **127**, 256403 (2021).
